# Physiological and Pathological Observations Connected with the Effects of Alcoholic Drinks upon the Liver

**Published:** 1855-11

**Authors:** E. Hillyer

**Affiliations:** Atlanta, Ga.


ARTICLE III.—*Physiological and Pathological Obeservations connected with the effects of Alcoholic Drinks upon the Liver.* By E. HILLYER, M. D., Atlanta, Ga.

There is, perhaps, no organ in the human body which suffers more from the excessive use of alcoholic drinks than the liver. Every physician has noticed that the drunkard, who is cooling off from a spree, has indications of great biliary derangement.

The physiological action of this organ, as now understood in the institutes of medicine, shows that it is perhaps more exposed to the deleterious effects of the stimuli than any other organ. Let us see what are its principal functions and uses to the animal economy, and then notice the effect which is produced by its derangement.

1. Its relation to the stomach and intestines is such that all drinks and nutritive fluids which enter them and are not taken up by the lacteals, must be absorbed by the blood vessels. These come together to form the Vena Portæ, the large trunk which extends along the groove of the liver. Upon the fluids which are thus brought through the liver from the stomach and intestines, it exercises a depurative and assimilating action. This is thought by many physiologists to be one of the principal and most important functions of this organ.

2. It has an excreto secretive action, as it separates from the blood those hydrocarbonaceous products, the result of disintegration of such tissues as cannot be formed into sugar or fat, to be burnt off by the union of them with the newly oxygenated blood. This excrementitious material is thrown into the duodenum from the liver, in connection with other bilious matter, formed from the different kinds of blood with which this gland is supplied.

3 The bile thus poured into the alimentary canal may assist digestion, first, by converting starchy matter into saccharine; secondly, uniting with the pancreatic fluid and assisting in emulsifying the facts and oils of the chyme; thirdly it acts as a stimulant to the peristaltic action of the bowels, thereby exercising a purgative influence upon them.

4. The liver has the power of eliminating from the blood of the portal veins saccharine matter in considerable quantity, which has received the name of liver sugar. This is readily susceptible of oxydation, and passes off in the blood of the hepatic veins, and is supposed to be one of the principal materials which are made subservient to the evolution of caloric in the process of combustion which is known to take place in the respiratory and circulatory apparatus.

This theory of elimination of sugar from, or the conversion of amylaceous matter in the portal veins into sugar, is given in the very elaborate observations of M. BERNARD upon the liver. His opinions are generally received with great weight by our most distinguished physiologists. Let us see what effect the continued action of alcohol will have upon the liver. It is brought by the portal circulation to the liver in an almost pure state, which, in its depurative and assimulative action makes an effort to modify and so change it, so that it will be rendered comparatively inert in the general circulation. But the liver is overtasked, called upon to perform more than it can accomplish, The portal veins are completely filled up and engorged with the fluids taken from the alimentary canal, the consequence is hyperemia or congestion of the liver. From this condition many diseases may be induced—hepatitis, acute and chronic; jaundice, ascites and general anasarca. These are most frequent; but there are a number of others which have their origin from the same cause. There is no difficulty in perceiving that this state of engorgement will very likely bring on inflammation in some form. From the long-continued effects of the stimuli upon its structure, softening is induced, so that it is impossible to detach it from its place without tearing it into shreds. The substance of the gland appears to be reduced to a kind of pulp, without any semblance of organization, and the whole mass of a purple or brown color. When the liver is reduced to such a state of disorganization, ascites must necessarily ensue. The hepatic extremities of the portal veins are occluded, often many of them obliterated. Their capillaries, which are distributed over the surface of the stomach and intestines, go on performing their function of absorption of fluids taken in as drinks and nutriment. These portal veins become engorged—filled up to such an extent that they are much increased in size—and it is impossible for them to contain their contents, and from their mesenteric and peritoneal surfaces the action of exosmose begins, by which they are relieved of their overburden, and we have a rapid accumulation of the watery portion of their blood in the cavity of the abdomen. This is a very frequent result from a whisky liver. From this disorganization of structure, jaundice is often an accompaniment of the ascites. We can conceive that it will most likely supervene, no matter which theory of Pathology we adopt as to the manner in which bile gets into the tissues, supposing that of DARWIN, M. CHEVERAL and MAYO, to be correct: that there is an excess of the eliments of bile in the blood, or that the liver is not adequate to the separating or eliminating it from the blood, and that it is precipitated into the skin and areolar tissue.

We have the liver in this condition that it is impossible for it to clear the blood of its bilious elements.

Then grant that the theory of reabsorption is correct. We have often the obstruction of the hepatic ducts to cause the bile to be taken into the general circulation in that way.

The writer remembers to have seen a case while a student which was laboring under both ascites and jaundice. He had been the keeper of a bar for several years, and drank to great excess. He first became dropsical and then jaundiced. There is no doubt but that both were occasioned by his liver being brought into this state of hyperemia and subsequent softening. Recently there was another case came under his observation which was successfully treated by him, in which there was a general anasarca in connection with the ascites. This general dropsy was supposed to be brought on by the hypertrophied liver pressing upon the ascending vena cava, causing an obstruction to the upward flow of the blood; or it might have been occasioned by debility and want of tonic action in the blood vessels, which allowed the serum to percolate through them. He was treated by mercurial and opiates at night, followed the next day by a drastic cathartic and diuretic pywder.

This patient recovered entirely. His liver could not have been further advanced to a state of disorganization than to excessive engorgement.

The digestion must suffer more or less from this state of the liver, as the the bile has always been considered very essential to healthy digestion. The pancreatic secretion does not have the assistance of the bile in the conversion of amylacious nutriment into saccharine matter, or in emulsifying the fats and oils, so that they can be more readily absorbed by the lacteals.

There are many fluids taken into the stomach as food which are not fit to enter the circulation even after they have gone through the digestive apparatus. As was before mentioned, the liver has a depurative and assimilative action upon them. Now, this great function of the liver is much interfered with when it is in this state of hyperemia. These fluids cannot pass through it while it is so much condensed and its channels occluded, and thus the changing and purifying of the portal blood does not take place. Its refuse elements, which should be thrown off with other hydro carbons and excrementitious matter from the general circulation, in the form of bile, which should be converted into glyco-cholic and tauro-cholic acids—these acids, with other constituents of bile, as we have seen, assist in digestiont—these are capable of being reabsorbed, and are now rendered susceptible of oxydation, by which they are carried out of the system in the form of carbonic acid and water. None of this is done. These excrementitious materials remain in the system and prove the cause of great irritation. Their presence will probably be more frequently a cause of disease than any to which the body is liable to be exposed. And in conclusion we would remark that for physiological reasons alone, habitual abstinence from alcoholic liquors is the best rule that can be laid down, for the great majority of healthy individuals, the exceptional cases in which any real benefit can be derived from their use, being extremely few."

